# Molecular Pathogenesis of Hodgkin Lymphoma: Past, Present, Future

**DOI:** 10.3390/ijms21186623

**Published:** 2020-09-10

**Authors:** Marc Bienz, Salima Ramdani, Hans Knecht

**Affiliations:** 1 Division of Hematology, Department of Medicine, Jewish General Hospital, McGill University, Montreal, QC H3T 1E2, Canada; marc.bienz@mail.mcgill.ca; 2 Department of Medicine, McGill University, Montreal, QC H3T 1E2, Canada; salima.ramdani@mail.mcgill.ca

**Keywords:** classical Hodgkin lymphoma, Hodgkin cells, Reed–Sternberg cells, genetic instability, telomere dysfunction, shelterin, TRF2, Lamin A/C

## Abstract

Our understanding of the tumorigenesis of classical Hodgkin lymphoma (cHL) and the formation of Reed–Sternberg cells (RS-cells) has evolved drastically in the last decades. More recently, a better characterization of the signaling pathways and the cellular interactions at play have paved the way for new targeted therapy in the hopes of improving outcomes. However, important gaps in knowledge remain that may hold the key for significant changes of paradigm in this lymphoma. Here, we discuss the past, present, and future of cHL, and review in detail the more recent discoveries pertaining to genetic instability, anti-apoptotic signaling pathways, the tumoral microenvironment, and host-immune system evasion in cHL.

## 1. Introduction

Since its first depiction, our understanding of classical Hodgkin lymphoma (cHL) has evolved from that of a mysterious disease characterized by grossly disfigured cells to that of a lymphoma with a complex biology that may hold the key to its cure. In 2016, the World Health Organization published its latest classification of lymphoma in which cHL is subdivided into four histological subtypes: nodular sclerosis, mixed cellularity, lymphocyte rich, and lymphocyte depleted [1]. Unifying to the pathognomonic multinucleated Reed–Sternberg cells (RS-cells) in all subtypes is the loss of the B-cell markers CD19 and CD20 and expression of CD15 and CD30, their paucity among the total amount of cells within the affected tissue, and the presence of EBV positive and negative forms [1,2]. This aggressive disease, affecting children, adolescents, adults, and elderly, of poor prognosis when advanced at diagnosis or relapsing/refractory, has caught the imagination of researchers for nearly two centuries.

In this review, we present the landmark discoveries that paved the way to the modern understanding of cHL biology and aim to tell the story of the Hodgkin (H) and Reed–Sternberg (RS) cells using some of the most recent evidence in the field.

## 2. Review

### 2.1. The Past—From Disease to Lymphoma

The first description of Hodgkin’s disease (HD) is attributed to Thomas Hodgkin. In 1832, he published a case series of his observations as “Inspector of the Dead” of Guy’s Hospital where he describes the gross findings of seven patients with gland and spleen enlargement in “On Some Morbid Appearances of the Absorbent Glands and Spleen” [3]. Hodgkin was the first to recognize this disease entity as “a primitive affection of those bodies, rather than the result of an irritation propagated to them from some ulcerated surface or other inflamed texture” [3,4]. It was then Sir Samuel Wilks that coined the term “Hodgkin’s disease” in his paper “Cases of Enlargement of the Lymphatic Glands and Spleen (or Hodgkin’s Disease) with Remarks” in 1865 [5]. 

In 1867, Olivier and Ranvier first characterized giant cells that are today known as Reed-Sternberg cells, a pathognomonic finding to HD. The first drawings of these multinucleated large cells are attributed to Greenfield in 1878 and lead to the first histological criteria independently described by Carl Sternberg (1898) and Donna Reed (1902) [4,6]. The etiology behind HD was still a mystery for over a century as opinions diverged on whether it was an infectious, inflammatory, or malignant entity [7]. Hodgkin himself saw it as a “hypertrophy of the lymphatic system”, while others such as Sternberg, thought it was infectious, perhaps from Tuberculosis and even viruses [4]. With the identification of aneuploidy and clonality in RS-cells in the 1960s, it became clear that it was a malignant entity [4]. The development of monoclonal antibodies by Kohler and Milstein in 1975 led to the identification of CD30 positivity in H- and RS-cells [8,9,10]. Giant leaps in our understanding were made possible by the establishment and characterization of the first, L-428 [11,12], and subsequent Hodgkin cell lines [13]. Polymerase chain reaction (PCR) techniques combined with single-cell micro-dissection allowed to molecularly analyze the mononuclear H-cells and bi- or multinuclear RS-cells from diagnostic lymph node biopsies [14]. H- and RS-cells were revealed to be neoplastic clonal B-cells of germinal center (GC) origin in 98% of patients [8,15,16,17]. With this new evidence, the WHO classification’s designation was changed from Hodgkin disease (HD) into Hodgkin lymphoma (HL) in the late 20th century [18,19]. 

### 2.2. The First Hit

Two comprehensive, in-depth reviews dealing with the pathogenesis [20] of cHL and morphogenesis [21] of RS-cells were recently published, but the initial, early post-germinal center lymphoid precursor of the mononuclear H-cell is still ill-defined. The development of malignant H- and RS-cells can be traced down to early events that trigger healthy germinal center B cells to transform step by step into neoplastic cells [22,23]. In EBV infection, crippled GC B-cells with nonfunctional immunoglobulin heavy gene-rearrangements, unable to express CD40 and the B-cell receptor (BCR) to receive survival signals, are rescued through LMP1 and LMP2A expression [24]. Such cells may in vitro survive as clonal lymphoblastoid cell lines [24] or in vivo persist as very rare B-lymphocytes in the memory compartment [25]. The association between EBV and cHL and its role in tumorigenesis is well demonstrated. In the USA and Europe, EBV positivity is found in up to 50% of cHL cases [26] but nearly 100% of cases occurring in some countries of Africa and Asia [20] or associated with HIV infection [27] are EBV positive. To account for this difference, it has been hypothesized that fulminant EBV infections at a younger age, as often seen in countries of lower socio-economic-status or high infectious burden, may increase the risk of EBV positive cHL given a maturing or stressed immune system [20,28]. Other observations include environmental and ethnic factors [29].

The underlying mechanism by which EBV acts as an oncogene in cHL is thought to arise from the expression of EBER1, EBER2, EBNA1, LMP1, LMP2A, and LMP2B (latency type II) seen in EBV positive H- and RS-cells [30]. Some of these proteins were shown to prevent maturation of the naïve B-cells and, LMP1 in particular, to provoke tumorigenesis through multiple pathways [20,31]. LMP1 is a multifunctional oncoprotein known to cause constitutive activation of the NF-kB pathway, which leads to inhibition of apoptosis and provides the cells other survival benefits (see Section 2.4). LMP1 also attracts Th2 and regulatory T cells through chemokines, which also promote survival [32]. A hypothetical sequence of events from a healthy naïve B-cell to a cHL granuloma through EBV infection has been described by Klein et al. [33].

Two mechanisms have been hypothesized to explain the development of cHL in EBV negative cases. In the hit-and-run theory, parts of the EBV genome persist integrated into the host genome (the normal circular episomal localization lost after several rounds of cellular division) and viral gene expression is lost when analyzed by classical testing giving the impression of EBV negativity [34]. This theory, which dates back to 1996, remains devoid of definite evidence [35,36]. Alternatively, it was shown that the NF-kB activation cascade was essential for cHL development (see Section 2.4) [20,37] but this activation was detected in H-cell lines and H- and RS-cells, i.e., morphologically clearly identifiable tumor cells. Thus, the true nature of the “first-hit” involved in EBV negative lymphoid precursors cHL remains elusive. 

Though it has been known for a long time that LMP1 and cyclin A are involved in the formation of H- and RS-cells [38,39], the complex molecular pathways involved in the trans-formation of the early clonal lymphoid precursor [24,40] to malignant H-cells and the “enigmatic” bi- or multinucleated end-stage RS-cells in both, EBV positive and EBV negative cHL, are still tantalizing research challenges. The current scenarios are reviewed and include i) the impact of telomere dysfunction on genetic instability in cHL, ii) the constitutional activation of anti-apoptotic cellular pathways, iii) the role of the tumoral microenvironment, and iv) the mechanisms of immune evasion.

### 2.3. Tying up Loose Ends—Telomeres

Telomeres are nucleoprotein complexes positioned at the end of each chromosomal arm and act as the guardians of the genome. They are composed of 5”-TTAGGG-3” non-coding tandem repeat DNA sequences. The T-loop is a knot-like structure at the tip of the telomere to protect its loose end. To secure the loop, a 50-400 nucleotide long 3” protruding G-rich single DNA strand elegantly inserts itself between the more proximal two strands of telomeric sequences (D-loop) [41,42]. 

In 1982, shelterins, a protein complex enveloping the telomeres, were first described in ciliates [43]. We now know that the six shelterin proteins in humans (TRF1, TRF2, TIN2, POT1, TPP1, and RAP1) are crucial in protecting the telomeric structure and orchestrating the formation of the T-loop [41,44]. Shelterins also repress DNA damage repair (DDR) pathways to avoid unintentional telomeric alterations [41,44]. Telomerase is a reverse transcriptase able to prolong the 3’ telomeric end and slow down the process of telomere shortening. Its discovery in the 1980s was recognized by the reception of the 2009 Nobel Prize in Physiology of Medicine [45,46,47,48]. Telomerase activity was found to be higher in germline and stem cells and nearly absent in somatic cells, which explains the normally occurring telomeric shortening with each cell division [49,50]. In 1997, telomerase activity in cHL was detected for the first time by Brousset et al. [51] and Bryan et al. described the principle of alternative lengthening of telomeres (ALT), a telomerase-independent pathway able to maintain telomeric length in malignant tumors [52]. 

Telomeric alterations from dysfunctional shelterin complexes, extreme telomeric shortening, or telomeric lengthening are among the mechanisms by which telomeres are implicated in carcinogenesis and were recently reviewed [21,53,54].

The development of telomeric three-dimensional Q-FISH (3D Q-FISH) was successfully used to study 3D telomere structure, with the distinct advantage of accurately quantifying telomere size and nuclear distribution [55,56,57,58]. This enabled our group to better describe the complex telomeric remodeling that is involved in the transition between H- and RS-cells. These changes can be summarized as (1) altered nuclear telomere distribution and number, (2) increasing formation of telomere aggregates, (3) increasing formation of t-stumps, and (4) progressively disrupted 3D interaction with shelterin proteins [59].

Using EBV negative H cell lines and tissue samples, we first demonstrated with 3D technology that RS-cells had significantly shorter telomeres compared to H-cells [60]. These very short telomeres, so-called t-stumps, are the result of gradual telomeric erosion leading to DNA uncapping. Thus, the unprotected chromosomal arms can readily form end-to-end fusions of chromosomes and aberrant mitotic spindles. Fragile di-centric chromosomes risk breakage leading to a vicious circle of breakage-bridge fusion (BBF), a hallmark of genomic instability [60,61]. Such large chromosomal aggregates were more frequently encountered in RS-cells compared to H-cells. However, some nuclei in RS-cells were unique in that they were nearly deprived of telomeric signals (“ghost nuclei”) and were seen in the vicinity of nuclei rich in large aggregates and chromosomes containing repeated alternating sequences of two different chromosomes as a result of several mitotic BBF cycles. We called these chromosomes “zebra chromosomes” [61]. This was shown, using spectral karyotyping (SKY) and super-resolution microscopy, to be the result of unequal chromosome segregation and centrosome duplication defects leading to gradual aneuploidy [61]. Such “ghost nuclei” and very short telomere allowed us to identify LMP1-expressing RS-cells as end-stage cells [62]. In particular, a high percentage of t-stumps and aggregates already in mononuclear H-cells of tissue biopsies correlated with refractoriness to treatment and relapsing disease [63]. 

To further understand why telomere shrinkage occurred in cHL, our group studied the behavior of the shelterin protein TRF2 in H- and RS-cells of LMP1 positive and EBV negative cHL. Two patterns of the TRF2–telomere 3D interaction were identified [64]. In the first pattern, restricted to EBV-negative cHL cases, H- and RS-cells were associated with massive attrition of telomere signals and a considerably increased number of free TRF2 signals. In half of these cases, abnormal DNA bridges were observed [64]. It has been shown experimentally, that elevated TRF2 is associated with anaphase bridges and telomere shrinkage [65]. In the second pattern, the telomere-bound TRF2 signal was lost progressively from H- to RS-cells, leading to telomere deprotection. This pattern was typical of, but not restricted to, LMP1-positive cases. “Ghost cells”, void of both TRF2 and telomere signals, were increasingly observed [64] suggesting to trigger further ATM-mediated telomere damage [41]. In EBV negative H- and RS- cells, the exact mechanism by which TRF2 is down-regulated, is unclear but may involve downstream effectors such as miR-23 [66]. These findings demonstrate that 3D TRF2-telomere interaction is universally dysregulated in cHL and can lead to the described chromosomal changes from H- to RS-cells through two distinct key mechanisms [64].

The disorganization of chromosomes described in EBV negative Hodgkin cell lines [67] is experimentally inducible. We demonstrated that LMP1 expressed in an EBV negative post-GC B-cell line may strongly alter telomere dynamics [68]. In an LMP1 tet on/off system of stably transfected EBV-negative BJAB cells, constitutive LMP1 activation significantly increased the formation of multinuclear RS-like cells and was associated with a significant increase in short telomeres and aggregates as well as complex chromosomal rearrangements. These observations were more impressive after multiple rounds of mitosis by 2–3 weeks, at which point ghost-nuclei and aggregate-rich satellite nuclei were regularly identified. The generation of ghost-nuclei was confirmed by SKY [68]. The impressive chromosomal aberrations achieved by constitutive LMP1 expression lead to the confirmation of our hypothesis and demonstration that LMP1 interacts substantially with the shelterin protein complex. TRF2 levels decreased >70% 3 days after LMP1 induction and were reversed upon LMP1 suppression as demonstrated by rt-PCR and Western blot. TRF1 and POT1 were also reversibly down-regulated. The use of combined telomere FISH and TRF2 immunostaining further confirmed the loss of TRF2 signals [68]. The most impressive and easily identifiable sign of chromosomal dysregulation is multi-nucleation, the hallmark of the diagnostic RS-cell. In our experimental design, LMP1 induced TRF2 down-regulation was the key factor to lead to multi-nucleation [68]. Substantial experimental support of our findings was recently published by the group of Shannon C Kenney [69]. They demonstrated the formation of cHL-like tumors containing multi-nucleated RS-like cells with high LMP1 expression in a cord blood humanized mouse model infected with the EBNA2-deleted EBV strain P3HR1. In this experimental design, any putative trans-activating activity of the EBNA2 oncogene was excluded through P3HR1 strain selection and the RS-like cells were of latency type II phenotype (69). In 2017, using 3D TRF2/Telo-Q-FISH we proved that TRF2 directly bound to telomeres was shown to be lost (down-regulated) progressively during the transition from H- to RS-cells in LMP1-expressing tumor cells of cHL, leading finally to the formation of ghost cells [64,70]. Thus, proof of principle was obtained with diagnostic lymph node biopsy material.

**Figure 1:**
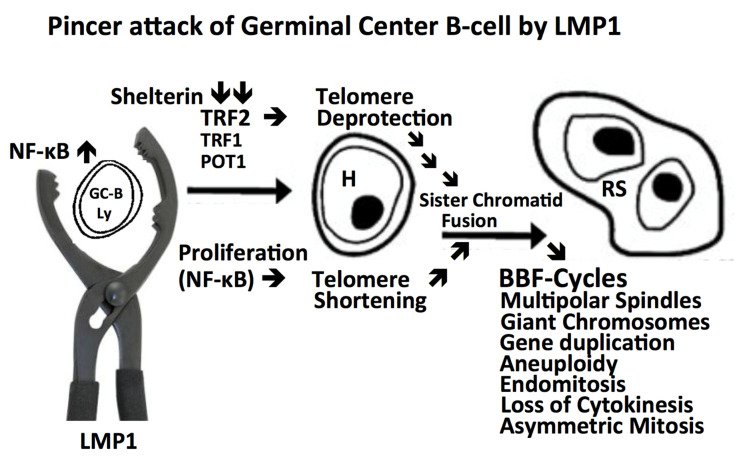
Continuous transformation from a GC B-lymphocyte into an H-cell and terminal RS-cell. A “crippled” GC B-lymphocyte [24] or an EBV+ memory B-lymphocyte [25] re-entering GC reaction undergoes LMP1 oncoprotein induced transformation targeting the telomere-shelterin complex through a “pincer attack”. NF-kB driven mitosis [37] leading to telomere shortening and TRF2 down-regulation [60,68] resulting in telomere de-protection, both in concert generate short de-protected telomeres, prone to undergo sister chromatid fusion as the origin of repeated BBF-cycles, resulting first in H-cells [60], and, after several subsequent mitotic cycles, in the end-stage multi-faceted, often telomere poor RS-cells [60,61,62,67,68,69] The telomere-shelterin complex appears to emerge as a logical candidate for a common pathogenic denominator of both EBV-positive and EBV-negative cHL because LMP1 alone induces multi-nuclearity also in the 293 embryonic kidney cell line [38] and the myelomonocytic HD-MyZ cell line [30].

The above-mentioned findings in both EBV negative and EBV positive cHL have convincingly demonstrated that the evolution of H-cells into RS-cells is orchestrated by a disruption in the shelterin protein complex which leads to telomere vulnerability, loss of size, disorganized fusions, and multi-centric chromosomes. These profound changes may be responsible for impaired chromosome migration, aneuploidy, and lead to the formation of multinucleated telomere-poor end-stage RS-cells. This scenario, starting with a GC B-lymphocyte expressing the LMP1 oncogene and ending up as a secretory highly active, cytokine-producing end-stage RS-cell, is depicted in Figure 1.

### 2.4. Intracellular Anti-Apoptotic Signaling Pathways

Three pathways, NF-kB, JAK-STAT, and MAPK/ERK, were shown to be activated in H- and RS-cells and involved in increased tumor cell survival.

The involvement of the NF-kB pathway in tumorigenesis is well documented [71,72]. The NF-kB family, composed of Rel-A, Rel-B, cRel, p50, and p52, is a group of cytoplasmic proteins that translocate to the nucleus and act as transcription factors for proteins involved in inflammatory and anti-apoptotic signaling cascades [73]. The activation of NF-kB is restricted by NF-kB inhibitors, which undergo ubiquitination and degradation upon activation of specific kinases. These kinases are regulated by the canonical and the non-canonical pathways. The canonical pathway is induced by inflammatory cytokines (namely TNF and IL-1) [71]. The non-canonical pathway is activated by ligands such as CD40L and RANKL and stimulates the kinase NIK (MAP3K14) [71]. Many mechanisms have been demonstrated to lead to NF-kB constitutional activation in Hodgkin lymphoma [73,74,75]. In EBV positive HL, LMP1 was shown to mimic CD40L and activate the non-canonical pathway [76]. Moreover, the microenvironment is a source of CD40L production. Hodgkin lymphoma has also been demonstrated to harbor mutations that amplify REL or inactivate NF-kB inhibitors such as TNFAIP3 and NFKBIE (Ikb) [76,77,78,79]. Such mutations are hypothesized to contribute to NF-kb constitutional activity in EBV negative cHL [33]. NF-kB nuclear translocation was shown to activate many genes known to play a role in cHL pathogenesis including IL-6, GM-CSF, and to prevent apoptosis in stress conditions [33,80].

The JAK-STAT signaling pathway requires activation of the JAK tyrosine kinases, which include JAK1, JAK2, JAK3, and TYK2 on the inner side of the cytoplasmic membrane. Each kinase is constitutively bound to a specific receptor such as the erythropoietin receptor, granulocyte colony-stimulating factor receptor, and interleukin receptors. When activated by the receptor’s specific ligand, the associated JAK kinase is activated [81]. These ligands are usually secreted by the cHL’s microenvironment [27]. This leads to phosphorylation of the cytoplasmic STAT transcription factors and translocation of STAT to the nucleus [82]. Target genes include namely those involved in proliferation (i.e., Cyclin D1, c-myc), survival (i.e., BCL-2), angiogenesis factors (i.e., VEGF) [83]. In addition to the constant stimulation from the tumoral microenvironment, intrinsic anomalies within the JAK-STAT pathway have been described in cHL that would lead to constitutive activation of the pathway. Amplification of the JAK2 9p24 locus was found in 30% of cases of cHL [84], and anomalies of the SOCS-1 protein, a member of the pathway’s negative feedback loop, were described both in cHL tissue and cell lines [85,86]. Moreover, in a study of the coding genome of cHL, using micro-dissected tumor samples, it was shown that 87% of cHL cases had dis-regulated JAK-STAT pathway as a result of multiple genetic alterations. In addition to confirming aberrant JAK2 and SOCS1, these alterations included activating mutations of JAK1, STAT3, STAT5B, and STAT6. Of particular interest, STAT3/STAT5B/STAT6 mutations were more prevalent in cases of concomitant SOCS1 inhibitor mutations [78].

The MAPK/ERK pathway involves receptor-linked tyrosine kinases such as the epidermal growth factor receptor, which leads to activation of RAS, RAK, and MEK. MEK is the protein responsible for phosphorylating and activating MAPK (previously known as ERK). Through many distinct pathways, the activated MAPK can regulate the activity of transcription factors such as MYC. Downstream upregulated genes include Cyclin D1, which is involved in cell proliferation. Aberrant activity of phosphorylated MAPK was demonstrated in cHL cell lines and tissue samples. An increased MAPK phosphorylation was achieved with CD30, CD40, and RANK receptor activation leading to increased cell survival [87].

Put together, there is growing evidence to support the fact that anti-apoptotic signaling pathways are constitutively activated in both EBV positive and negative cHL as a result of intrinsic mutations of key regulators of the pathway and from constant activation of its receptors from the tumoral microenvironment (Table 1, upper part).

### 2.5. The Tumoral Microenvironment

Unique to HL, the malignant cells seen (H- and RS-cells) represent <5% of cells in the affected tissue. The other residing cells are organized and form the tumoral microenvironment. This diverse tumoral ecosystem is composed of lymphocytes (CD4+ T-cells, plasma cells, B-cells), myeloid cells (eosinophils, macrophages, mast cells), and stromal cells (fibroblasts, mesenchymal stromal cells, endothelial cells) [88]. The microenvironment is not reactive to cancer but rather the result of a complex cytokine-mediated recruitment of healthy cells. These are later “indoctrinated” by H- and RS-cells to work to the tumor’s advantage to promote cancer cell survival, angiogenesis, and immune system evasion [88].

The mechanisms by which H- and RS-cells attract such a diversity of accomplices is mediated through cytokine and growth factor release. Examples include secretion of IL-5 to recruit eosinophils, CCL5, and M-CSF for macrophages, IL-7 for Treg lymphocytes, and FGF-2 for fibroblasts and endothelial cells. The list of all involved cytokines is reviewed by Aldinucci et al. [88]. 

T-cells are the most prevalent cells of the cHL microenvironment. The CD40L-positive CD4+ T-cells are often encountered around H- and RS-cells, hence their nickname of rosetting T-cells [89]. These cells, through CD40L, CD80, and CD54 can shield the H- and RS-cells from the adaptive immune system of cytotoxic T-cells and natural killer cells [88]. H- and RS-cells also can educate CD4+ T-cells to polarize towards T regulatory cells (Tregs), which also play a role of immunosuppression in the microenvironment [32,88,89].

Macrophages impact the microenvironment in an array of ways. Through secretion of M-CSF, H- and RS-cells can promote monocyte differentiation into M2 macrophages. M2 macrophages have a phenotype that favors tumor survival through angiogenesis promotion, tumor growth stimulation, and anti-inflammatory properties [88,90,91]. Fibroblasts, also, are not benign bystanders as they can be transformed under the guidance of H- and RS-cells in so-called cancer-associated fibroblasts through the secretion of vesicles that are internalized by fibroblasts. These specialized fibroblasts contribute to angiogenesis, extracellular matrix production, and growth factor secretion [88].

The complex intricacies of the tumoral microenvironment go beyond the scope of this paper and have been comprehensively reviewed by Carreau et al. and Aldinucci et al. [88,92]. However, it must not go unmentioned that this microenvironment has become a field of interest for targeted therapy such as PD-1 immune checkpoint inhibitors and our understanding of the cellular interactions will lead to more efficient treatments in the future [92] (Table 1, lower part).

### 2.6. Host-Immune System Evasion

Immune tolerance is an important mechanism by which H- and RS-cells promote their own survival. One mechanism of immune escape is the immunosuppressive effect of the microenvironment [27,93]. In fact, CD8+ T-cells require β2-microglobulin (B2M) and Major Histocompatibility Complex (MHC) class I antigens as targets to fulfill their role in the cytotoxic response of our immune system. However, in H- and RS- cells the expression of these molecules is down-regulated in 90% of cases [94]. Meanwhile, CD4+ helper T-cells require MHC class II to function but its expression is reduced or aberrant in 40% of cHL [95]. 

**Figure 2:**
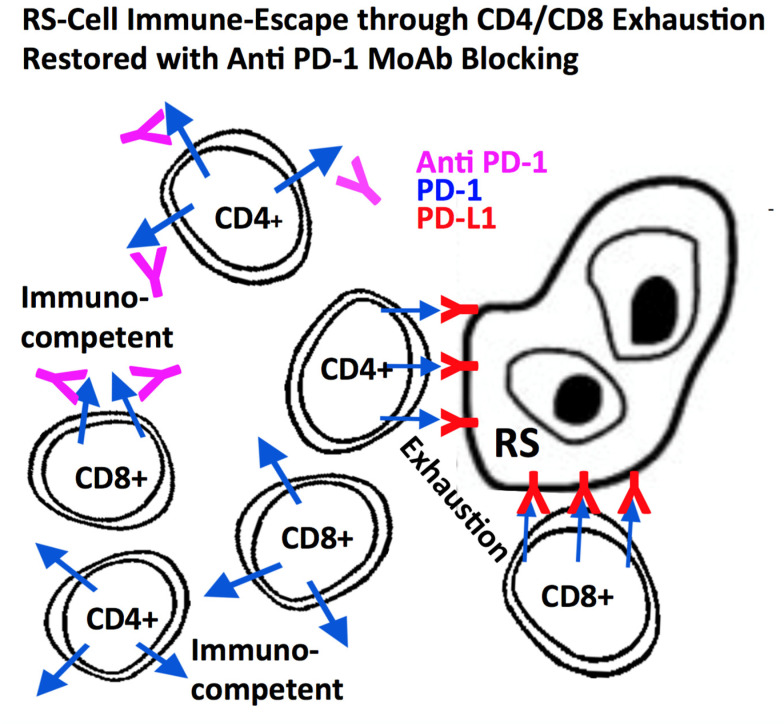
Hodgkin (H) and Reed-Sternberg (RS)-cells (especially LMP1+) express a high number of PD-L1 & PD-L2 molecules at their surface thereby paralysing normal CD8+ cytotoxicity and CD4+ cytokine secretion. MoAbs directed against PD-1, expressed on CD4+ and CD8+ lymphocytes, block PD-1 signaling thereby restoring CD8+ cytotoxic activity and CD4+ antigen recognition and effector function, when docking at the MHC class-I (CD8+) or MHC class II receptor (CD4+) at the surface of H- and RS-cells [96,97,98,99,100,101,102,103,104].

Despite these attempts to camouflage, cHL antigen presentation does occur. In these cases, through their immune checkpoint receptors, H- and RS-cells are able remain undetected. One prominent example is the programmed death 1 (PD-1) T-cell surface receptor which, when engaged by the ligand PD-L1 of the antigen-presenting cell (i.e., H- and RS-cells), induces transcription of genes aimed at suppressing its effector or cytotoxic functions [96]. This contributes to a state of “T-cell exhaustion” as activated T-cells are inhibited through PD-1 signaling [97]. Several mechanisms can lead to high expression of PDL-1 in cHL such as amplification of the 9p24 chromosomal section, seen in a third of cHL cases [98]. This chromosomal region expresses the PD-L1 gene locus. It also promotes PD-L2 and Janus kinase 2 (JAK2) expression, both upregulating PD-L1 expression on the cell surface, partly through JAK/STAT signaling [96,99]. In EBV positive HL, EBV is also involved in host-immune system evasion by activating the AP1 transcription factor as well as up-regulating c-Jun and JunB, thereby increasing PD-L1 and PD-L2 independently of the 9p24.1 copy number [96,99,100]. The understanding of the importance of PD-L1 in this disease has led to the use of targeted PD-L1 inhibitors in cHL to enhance T-cell function [101] (Figure 2). Results have been very encouraging with responses generally correlating with the intensity of PD-L1 expression and amplification of the chromosome arm 9p24.1 [96]. The advent of PD-L1 inhibitors has been one of the recent great success stories of therapeutics in oncology [102,103,104].

Other immune checkpoints at play and contributing to T-cell exhaustion are CTLA-4, TIM3, 2B4, LAG-3, CD160, BTLA, and CD112R/TIGIT [96,103,104]. CTLA-4 inhibitors and chimeric antigen receptor (CAR) T cell therapies are being studied in relapsed/refractory cHL with encouraging early results, but severe adverse effects, mainly immune-related toxicities are a major issue [103,104].

### 2.7. The Future

Refined, extended clinical and pathologic staging, including detailed family history, screening for B-symptoms (fever, night sweats, weight loss), specific inflammatory laboratory parameters, PET-CT staging, and a diagnostic lymph node biopsy (including a complete lymph node) are primordial for a risk-stratified optimal therapeutic approach [103]. Lymph node core biopsies, though less invasive for the patient, are much less informative and are associated with the risk of sampling errors. A detailed immunopathologic diagnosis, including the lymph node architecture, percentages of infiltrating CD4+ and CD8+ lymphocytes, tumor-associated macrophages, and eosinophils, belongs to an optimal work up. This interactive (interdisciplinary) diagnostic approach between hemato-pathologists, nuclear medicine specialists, and treating hematologists/oncologists, has drastically increased the predictability of long-term survival of this particular B-cell lymphoma [103,104]. The next decade will teach us if cHL will be the first aggressive B-cell lymphoma with a cure rate near to 100%. 

Even in the area of new immune-mediated therapies refractory/relapsing cHL remains a major issue [104,105]. The 5-year survival rate for cHL is currently around 87% [105]. Further improvement of this percentage close to 100% is only possible if researchers arrive to further characterize the small lymphoid precursor cells in both, EBV-associated and non-associated cHL. These currently unidentified precursors are silent for months to years (late relapses) or permanently active (cycling) in refractory cHL. 

**Figure 3:**
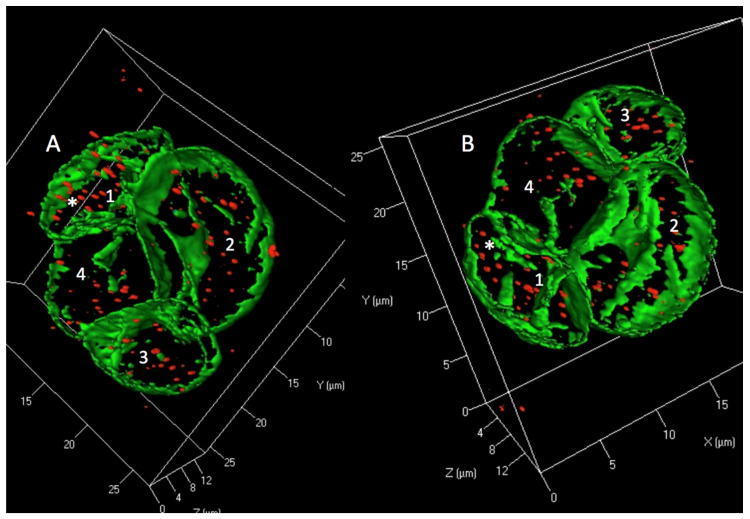
Tetra-nuclear (nuclei 1–4) Reed–Sternberg cell of the Hodgkin cell line HDLM-2 is shown in 3D surface mode with lamin A/C in green and TRF2 in red. Impressive lamin A/C intranuclear septa are seen. TRF2 signals are completely separated from lamin A/C indicating a major disturbance of nuclear architecture. (**A)**. Asterix (*) points to a region where at first glance a few TRF2 signals appear to co-localize with lamin A/C. (**B)**. However, turning the RS-cell 180° in the z-axis and about 30–40° in the y- and x-axis, clearly reveals separation (*) of lamin A/C and TRF2. This is consistent with a major disturbance of the lamin A/C – TRF2 interaction.

Applying a new form of mass cytometry, the so-called “multiplex single-cell morphometry” based on the identification of a dozen of intracellular molecules related to structure and function (including lamin A/C, beta-actin, lysozyme, the vesicle docking molecule VAMP-7), Bendall and coworkers [106] demonstrated distinct morphometric markers for each major hematologic cell type. Interestingly, lamin A/C helped to distinguish normal from mature neoplastic T-cells (T-PLL). We recently demonstrated distinct 3D structural lamin A/C expression patterns in H and RS-cells [107] earlier identified with 2D immunohistochemistry [108]. Knowing that the nuclear 3D structure is related to the direct interaction of TRF2 with lamin A/C [109] and that loss of lamin A function increases chromatin dynamics in the nuclear interior [110] (Figure 3), it might be worth to investigate the lymphocyte population of Hodgkin lymphoma lymph nodes by “multiplex single-cell morphometry” [106]. Though most of the lymphocytes surrounding H- and RS-cells are T-lymphocytes, B-lymphocytes are also present and few lymphocytes express lamin A/C in immunohistology. Are these the H and RS-cell precursors?

The cell nucleus undergoes during EBV infection reprogramming of the nuclear architecture, DNA replication, and histone deposition [111]. Both EBV and HHV-6, target the nucleus at the telomeres. Whereas LMP1 acts indirectly via shelterin down-regulation [68,70], HHV-6 directly integrates the telomeric region through viral hexanucleotide telomeric TTAGGG repeats, identical to those of human telomeres [112,113,114] and HHV-6 was identified by immunohistochemistry, single-cell PCR, or FISH in RS-cells of 50% of cHL biopsies [115]. Based on these findings, the 3D interaction of the shelterin proteins with HHV-6 may be worth further investigation. A further, recently discovered protein, TZAP (telomeric zinc finger-associated protein) is a direct competitor of TRF1 and TRF2, binding preferentially to long telomeres [116]. Is TZAP involved during EBV infection of precursor lymphocytes by replacing down-regulated TRF1 and TRF2?

As the 3D cancer cell nucleus is a dynamic structure whose end-stage alterations are mostly identifiable [117] it is mandatory to detect and to analyze the very early changes on the road to malignancy with recently developed super-resolution microscopic techniques [118], humanized mouse model systems [69], and mass cytometry of nuclear structural proteins [106].

## 3. Conclusions

Throughout this review, we have described multiple aspects of the tumorigenesis of classical Hodgkin lymphoma from the first genetic insults on healthy germinal center B-cells to their development into RS-cells through genetic instability orchestrated by telomeric dysfunction. We have also discussed the multiple ways by which H- and RS-cells ensure their survival with the help of a selective recruitment of cells in their microenvironment, the constitutional activation of anti-apoptotic pathways, and elaborate host-immune system evasion. Our better understanding of this lymphoma has allowed us to demystify the puzzling RS-cells and to substantially improve treatments cure rates and survival in relapsing/refractory cases. Nevertheless, the complete understanding of cHL remains challenging and will only be achieved through research focused on the early “dormant” lymphoid precursor and the complete molecular deciphering of its transition to H- and finally RS-cells. It is the missing final step to cure cHL.

## Figures and Tables

**Table 1:** Review of mechanisms involved in classical Hodgkin lymphoma (cHL) enhanced cell survival, apoptosis resistance, and proliferation.

**Intracellular Anti-Apoptotic Signaling Pathways**	
**Trigger of Pathway Activation**	**Resulting Pathway Involved**
LMP1 oncoprotein activation	Constitutive activation of the NF-KB signaling pathway
CD40L expressed by eosinophils recruited and residing in the tumoral microenvironment	
Acquired mutations inactivating NF-KB inhibitors	
LMP2A activation	Constitutive activation of the JAK-STAT signaling pathway
Secretion of JAK-STAT’s specific ligands by the tumoral microenvironment	
Acquired activating mutations within JAK-STAT’s signaling cascade	
Aberrant activity of phosphorylated MAPK	Constitutive activation of the MAPK/ERK signal pathway
**Tumoral Microenvironment**	
**Trigger of Cell Activation**	**Resulting Cell Involved**
M-CSF secreted by HL cells, endothelial cells and fibroblasts promotes macrophage differentiation to M2-polarized macrophages.	M2-polarized macrophages
Extracellular vesicles secreted by cHL cells are internalized by fibroblasts leading to a switch of phenotype	Cancer-associated fibroblasts (IL-6 secretion)
